# Supplementary figures and images for: Metabolic memory of Δ9-tetrahydrocannabinol exposure in pluripotent stem cells and primordial germ cells-like cells
Source: eLife. 2023 Dec 27;12:RP88795. doi: 10.7554/eLife.88795 (PMC10752584; doi:10.7554/eLife.88795)

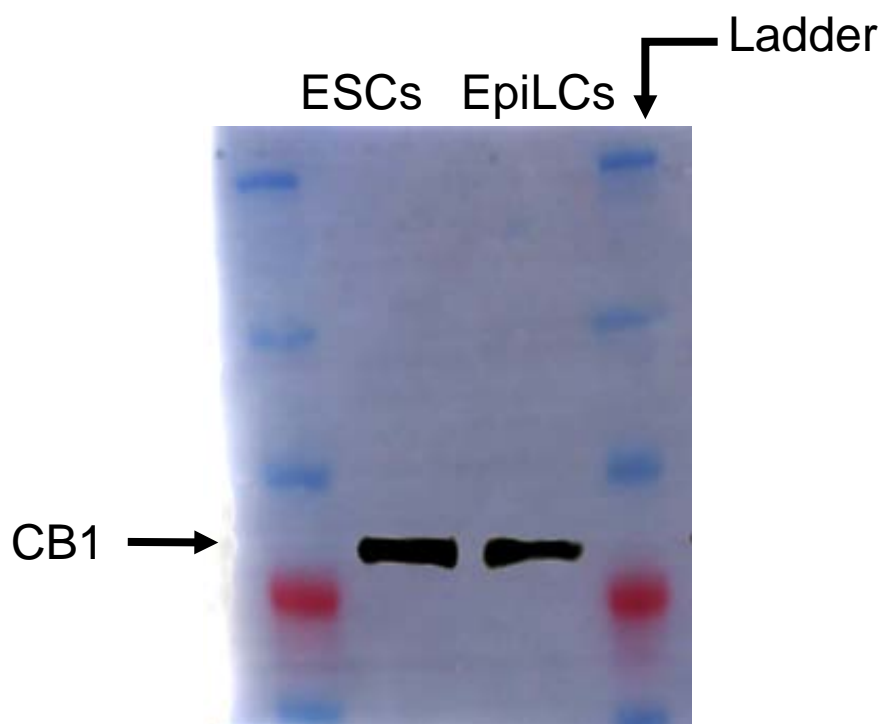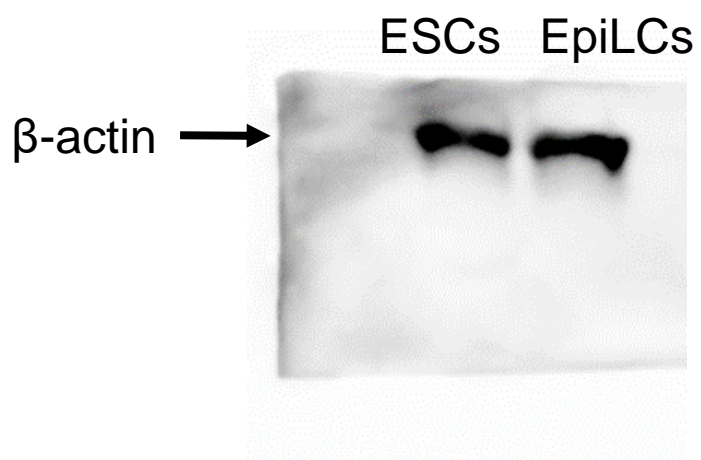

Supplement: Figure 2—source data 1. [file elife-88795-fig2-data1.pdf]
